# Special Issue “Natural Compounds: Advances in Antimicrobial Activity”

**DOI:** 10.3390/ijms262311317

**Published:** 2025-11-23

**Authors:** Carlo Genovese, Francesco Pegreffi

**Affiliations:** 1 Department of Medicine and Surgery, School of Medicine and Surgery, University of Enna “Kore”, 94100 Enna, Italy; francesco.pegreffi@unikore.it; 2 Recovery and Functional Rehabilitation Unit, Umberto I Hospital, 94100 Enna, Italy

The global escalation of antimicrobial resistance represents a critical threat to public health, agriculture, and clinical medicine [1]. Conventional antibiotics are increasingly compromised by multidrug-resistant pathogens, necessitating the exploration of alternative therapeutic agents [2]. Advances in metabolomics, cheminformatics, and high-throughput screening have accelerated the discovery pipeline, enabling the rapid identification of lead compounds and the optimization of their pharmacokinetic and pharmacodynamic profiles [3]. Moreover, the integration of molecular docking, in silico modeling, and structure-activity relationship analyses has facilitated the rational design of analogs with enhanced potency and selectivity [4].

Bioactive compounds derived from plants, microorganisms, marine organisms, and other biological sources represent a prolific reservoir of structurally diverse molecules with potent antimicrobial properties [5]. Naturally derived agents, long revered for their pharmacological potential, are once again at the forefront of scientific inquiry, offering promising avenues to combat resistant pathogens and restore the efficacy of antimicrobial treatments [6]. Beyond therapeutic applications, natural antimicrobials are increasingly investigated for their utility in food preservation [7], crop protection [8], and environmental remediation [9], underscoring their multidisciplinary relevance.

The Special Issue “Natural Compounds: Advances in Antimicrobial Activity” consolidates recent progress in the identification, characterization, and mechanistic elucidation of bioactive natural products with antimicrobial efficacy. The contributions herein span a broad spectrum of chemical compounds, including alkaloids, terpenoids, polyphenols, peptides, and glycosides, each exhibiting distinct modes of action such as membrane disruption, inhibition of nucleic acid synthesis, modulation of efflux pumps, and quorum-sensing interference.

In this Special Issue, several articles emphasize the synergistic potential of natural compounds when co-administered with conventional antibiotics, offering a promising strategy to overcome resistance phenotypes and restore antimicrobial susceptibility. Others explore the role of natural products in targeting biofilm-forming pathogens, a major challenge in chronic infections and medical device-associated contamination [10], as these resilient microbial communities exhibit heightened tolerance to conventional antibiotics and often necessitate innovative therapeutic approaches [11].

In this context, Campoccia et al. provide a comprehensive evaluation of KSL, KSL-W, and Dadapin-1 peptides, highlighting their cytocompatibility and selectivity profiles across multiple cell lines, and underscoring their potential for orthopedic anti-infective biomaterial applications [12]. In a further article, Campoccia et al. identify KSL-W as the most robust antimicrobial peptide for orthopedic applications, demonstrating superior bactericidal and anti-biofilm activity under physiologically relevant conditions [13]. Within the scope of antimicrobial and anti-biofilm research, other authors have advanced natural approaches, thereby broadening the spectrum of strategies under investigation. Specifically, Haj-Yahya et al. demonstrate that trans, trans-farnesol significantly enhances the antimicrobial and anti-biofilm activity of arachidonic acid against *Streptococcus mutans* and *Streptococcus sobrinus*, offering a synergistic natural approach for caries prevention [14].

Beyond oral health, natural compounds have also been investigated for their relevance in dermatological contexts. Na Nongkhai et al. demonstrate that rhizome extracts of *Alpinia galanga* and *Zingiber zerumbet* possess potent antimicrobial activity against acne-associated bacteria, with low cytotoxicity and rich phytochemical profiles, underscoring their potential for dermatological applications [15].

Among membrane-targeting strategies, noteworthy contributions have been provided by Feng et al. and Tarek et al., who investigate distinct natural and protease-based agents capable of compromising bacterial integrity. Feng et al. report the first comprehensive evidence of the antibacterial efficacy of *Taxillus chinensis*, identifying 4-indolecarbaldehyde as a membrane-disruptive compound with broad-spectrum activity, including potent effects against methicillin-resistant *Staphylococcus aureus* [16]. In their mechanistic investigation, Tarek et al. demonstrate that protease SH21 from *Bacillus siamensis* exerts potent antimicrobial effects by compromising bacterial membrane integrity, highlighting its potential as a thermally stable, pH-tolerant candidate for combating resistant infections [17].

In addition to prior studies focused on human health, recent investigations have extended antimicrobial research to the veterinary domain, where bacteriocins are explored as natural alternatives to conventional antibiotics in poultry farming. Notably, Mamjoud et al. demonstrate that bacteriocins, such as microcin J25 and pediocin PA-1, can modulate chicken cecal microbiota with minimal disruption to microbial diversity, offering promising alternatives to conventional antibiotics in poultry farming [18].

The contributions gathered in this Special Issue underscore the remarkable diversity and translational potential of natural and synthetic antimicrobial compounds. From plant-derived molecules and bacteriocins to engineered peptides, each study offers mechanistic insights and application-oriented evidence supporting their role in combating microbial resistance and biofilm-associated infections.

Several works highlight membrane-targeting strategies as a recurrent and effective mode of action, while others emphasize the importance of cytocompatibility, selectivity, and environmental stability, critical parameters for clinical and industrial deployment. The comparative evaluations of antimicrobial peptides for orthopedic applications and the microbiota-sparing effects of bacteriocins in poultry models exemplify the multidisciplinary relevance of this research.

Together, these findings reinforce the value of natural compounds and peptide-based therapeutics as viable alternatives to conventional antibiotics, with promising implications for medicine, agriculture, and biotechnology. We hope this collection will stimulate further innovation and collaborative efforts in the development of next-generation antimicrobial solutions.
